# Venous Thromboembolism (VTE) Risk Assessment in Acute Inpatient Mental Health Wards in Sherwood Oaks and Millbrook Unit (now Blossomwood Unit), Nottinghamshire Healthcare NHS Foundation Trust

**DOI:** 10.1192/bjo.2025.10650

**Published:** 2025-06-20

**Authors:** Chinenye Omesili, Farah Bashir

**Affiliations:** 1 Nottinghamshire Healthcare NHS Foundation Trust, Rampton, United Kingdom; 2 Nottinghamshire Healthcare NHS Foundation Trust, Mansfield, United Kingdom

## Abstract

**Aims:** To assess compliance with the trust policy and NICE guidelines on VTE risk assessment for new admissions into the acute psychiatric wards in Millbrook and Sherwood Oaks mental hospitals, Nottinghamshire NHS Foundation Trust.

**Methods:** A retrospective audit looked at case notes of patients aged 20–80 years, admitted within a 2 weeks period across 8 wards in April 2023. This was re-audited in April 2024 after all recommendations were actioned. Infornation was collated and manually analysed. Data collected included but not exclusive to date of admission, date VTE risk assessment was done and the level of VTE risk identified. These were compared with the standard criteria which were the trust policy 02.21 – 'Patients who are admitted should have VTE risk assessment within 24 hours of admission’ and the NICE guidelines NG (82) 2019 – 'Assess all acute psychiatric patients to identify their risk of VTE and bleeding as soon as possible after admission to hospital or by the time of the first consultant review'.

**Results:** The first cycle found that only 69.3% of the patients admitted were assessed on admission (with 50% assessed within 24 hours of admission) whereas 30.7% were not assessed throughout the duration of their admission. The second audit cycle showed remarkable improvements. 80.5% were assessed for VTE risk (63.9% within 24 hours of admission) whereas 19.5% were not assessed.

The level of risk was categorized into low, moderate and high risk using Well’s scoring system. 69% of patients who were assessed in the first cycle, had low risk but risk of 31% of the cohort of patients audited were unknown because they were not assessed. In the second cycle,80.5% had low risk whereas 19.5% of the patients fell under the unknown category due to not having been assessed.

**Conclusion:** The importance of VTE risk assessment in acute inpatient wards can never be overemphasized. Studies show that psychiatric inpatients are likely to be at an increased risk of VTE due to – use of psychotropic agents, reduced mobility, dehydration as a result of self-neglect or suicidal attempts, prolonged restraints, sedation, co-morbid physical health problems etc.

There are still lapses in our patient management that need to be considered in order to provide an outstanding patient care and safety.

